# Is lifestyle change around retirement associated with better physical performance in older age?: insights from a longitudinal cohort

**DOI:** 10.1007/s10433-021-00607-9

**Published:** 2021-05-27

**Authors:** Sian M. Robinson, Leo D. Westbury, Kate Ward, Holly Syddall, Rachel Cooper, Cyrus Cooper, Avan A. Sayer

**Affiliations:** 1 AGE Research Group, Newcastle University Institute for Translational and Clinical Research, Newcastle upon Tyne, NE4 5PL UK; 2 NIHR Newcastle Biomedical Research Centre, Newcastle University and Newcastle Upon Tyne NHS Foundation Trust, Newcastle upon Tyne, UK; 3 MRC Lifecourse Epidemiology Unit, University of Southampton, Southampton, UK; 4 Department of Sport and Exercise Sciences, Musculoskeletal Science and Sports Medicine Research Centre, Manchester Metropolitan University, Manchester, UK; 5 NIHR Southampton Biomedical Research Centre, University of Southampton and University Hospital Southampton NHS Foundation Trust, Southampton, UK; 6 NIHR Oxford Biomedical Research Centre, University of Oxford, Oxford, UK

**Keywords:** Lifestyle, Prevention, Retirement, Physical function, Ageing

## Abstract

A growing evidence base links individual lifestyle factors to physical performance in older age, but much less is known about their combined effects, or the impact of lifestyle change. In a group of 937 participants from the MRC National Survey of Health and Development, we examined their number of lifestyle risk factors at 53 and 60–64 years in relation to their physical performance at 60–64, and the change in number of risk factors between these ages in relation to change in physical performance. At both assessments, information about lifestyle (physical activity, smoking, diet) was obtained via self-reports and height and weight were measured. Each participant’s number of lifestyle risk factors out of: obesity (body mass index ≥ 30 kg/m^2^); inactivity (no leisure time physical activity over previous month); current smoking; poor diet (diet quality score in bottom quarter of distribution) was determined at both ages. Physical performance: measured grip strength, chair rise and standing balance times at both ages and conditional change (independent of baseline) in physical performance outcomes from 53 to 60–64 were assessed. There were some changes in the pattern of lifestyle risk factors between assessments: 227 (24%) participants had fewer risk factors by age 60–64; 249 (27%) had more. Reductions in risk factors were associated with better physical performance at 60–64 and smaller declines over time (all *p* < 0.05); these associations were robust to adjustment. Strategies to support reduction in number of lifestyle risk factors around typical retirement age may have beneficial effects on physical performance in early older age.

**Supplementary Information:**

The online version of this article (10.1007/s10433-021-00607-9) contains supplementary material, which is available to authorized users.

## Introduction

Global increases in life expectancy, and the associated growth of older populations, have focussed international attention on strategies to promote health in later life (England and Azzopardi-Muscat 2017). There is particular interest in the role of lifestyle: factors such as diet and physical activity have been identified as key determinants of population health (McGinnis et al. 2002; Newton et al. 2015) and are thought to make an important contribution to observed heterogeneity in the ageing process (Lowsky et al. 2014). While the impact of poor lifestyle behaviours on the health of older adults may have received less attention than effects in younger groups (Rizzuto and Fratiglioni 2014) there is evidence to suggest that intervention to change these modifiable behaviours in later life could be an effective approach to promote health in older age (Rizzuto and Fratiglioni 2014).

Central to healthy ageing, and an ability to maintain independence in older age, is the preservation of an individual’s physical capability, with simple assessments of physical performance, including measures of muscle strength and function, shown to act as biomarkers of ageing (Cooper et al. 2010). There is increasing evidence of links between individual lifestyle ‘risk factors’ (such as obesity, physical inactivity, smoking, and poor diet) and poorer physical performance (Cooper et al. 2011; Dodds et al. 2013; Robinson et al. 2018; Stenholm et al. 2012). Importantly, health behaviours often cluster in individuals (Buck and Frosini 2012; Poortinga 2007) and graded increases in risk of poor performance are seen as the number of lifestyle risk factors increases. Described, both in cross-sectional (Robinson et al. 2013) and longitudinal (Cooper et al. 2016; Sabia et al. 2012) data from older populations, and linked to substantial differences in physical performance, these findings point both to an opportunity to use lifestyle change to protect the physical capability and health of older adults, but also to the need for further exploration to understand this potential.

Although the challenges of changing lifestyle are widely recognised, there may be particular opportunities to intervene in the period around retirement from work. Whilst the impact of retirement on individuals’ health behaviours may vary, with differences reported according to background characteristics including previous occupation, gender and other health behaviours (Baxter et al. 2016; Myllyntausta et al. 2017; Pulakka et al. 2019; Ali-Kovero et al 2020), it is recognised as a transition point in the lifecourse—with the pre-retirement period highlighted as a possible time for preventive actions (Zantinge et al. 2014). To examine lifestyle changes occurring in the period around typical retirement age, we analysed data from a longitudinal cohort study in which adults were characterised at the ages of 53 and 60–64 years. Our primary focus was on lifestyle change between these ages, with the hypothesis that reduction in lifestyle risk factors would be associated with better physical performance (grip strength, chair rise and standing balance) in early older age.

## Methods

### Study sample

The MRC National Survey of Health and Development (NSHD) is a longitudinal study based on a socially stratified sample of 5362 births occurring in one week in March 1946 across England, Wales and Scotland (Kuh et al. 2011). Data used in the present analyses (2019–2020) are from the follow-ups carried out in 1999 and 2006–2010. By the 2006–2010 follow-up, 718 participants had died, 594 had withdrawn from the study, 567 had emigrated and 320 were lost to follow-up. Of those remaining, 2229 (78% of those invited) were assessed: 1690 (76%) at clinic and 539 (24%) at home (Hurst et al. 2013; Kuh et al. 2011).

### Characterisation of lifestyle at 53 and 60–64 years

Detailed information on health and lifestyle was collected, and anthropometric measurements were made (Kuh et al. 2011).

(i) *Anthropometry*: Height and weight were measured using standard protocols by trained nurses and used to derive body mass index (BMI; kg/m^2^).
(ii) *Leisure time physical activity*: Participants were asked to report how often they had participated in any sports, vigorous leisure activities or exercise in their spare time in the previous month. Responses were categorized as follows: inactive (no participation); moderately active (participated in relevant activities 1–4 times per month); and active (participated in relevant activities 5 or more times per month (Dodds et al. 2013)).
(iii) *Smoking status* (never/ex/current) was categorized into current smokers and ex/never smokers.
(iv) *Diet quality*: Participants completed 5-day food diaries at both ages (Pot et al. 2015). Principal component analysis was used to identify dietary patterns, as reported previously (Robinson et al. 2018). The first component described a ‘healthier’ profile of foods, characterized by greater consumption of fruit, vegetables and wholegrain cereals. Participants’ scores for this pattern, indicating their compliance with it, were interpreted as measures of their diet ‘quality’.

The number of lifestyle ‘risk factors’ was determined for each participant at 53 and 60–64 out of: obesity (BMI ≥ 30 kg/m^2^); inactivity (no leisure time physical activity over the previous month); smoking (current); poor diet (diet quality score in bottom quarter of the distribution defined at 60–64). The total number of lifestyle risk factors ranged between 0 and 4 at each age; the change in number for each participant was determined by subtracting the number at 53 from the number at 60–64: positive scores indicated a less healthy lifestyle by age 60–64; negative scores indicated a more healthy lifestyle.

History of diabetes and cardiovascular disease was determined from self-reports of diabetes and doctor-diagnosed angina and myocardial infarction up to and including age 60–64. Participants reported whether they were in paid work at each age, including self-employment.

### Physical performance outcomes

At both ages, physical performance was assessed by trained nurses following standardised protocols. Grip strength was measured twice in each hand at 53 and three times in each hand at 60–64 using a Nottingham electronic handgrip dynamometer. The highest value at 53 and the highest of the first four values at 60–64 were used as in a previous analysis (Cooper et al. 2016). The time taken to perform 10 chair rises (rise from a sitting to a standing position and sit back down again) was recorded and used to derive chair rise speed as the number of repetitions per minute. Standing balance time was measured as the length of time a participant could stand on one leg with their eyes closed, up to a maximum of 30 s.

### Statistical analysis

Data were described using means and standard deviations, medians and interquartile ranges and frequency and percentage distributions. We limited our analyses to include participants for whom there were complete data on all lifestyle risk factors. Of the 2229 participants assessed at age 60–64, 1292 were excluded from the analyses as their data on diet (*n* = 914), other lifestyle factors, physical performance outcomes or potential confounders (*n* = 378) were incomplete (shown in Supplementary Fig. 1). The study population for the present analyses included 937 participants.

**Fig. 1:**
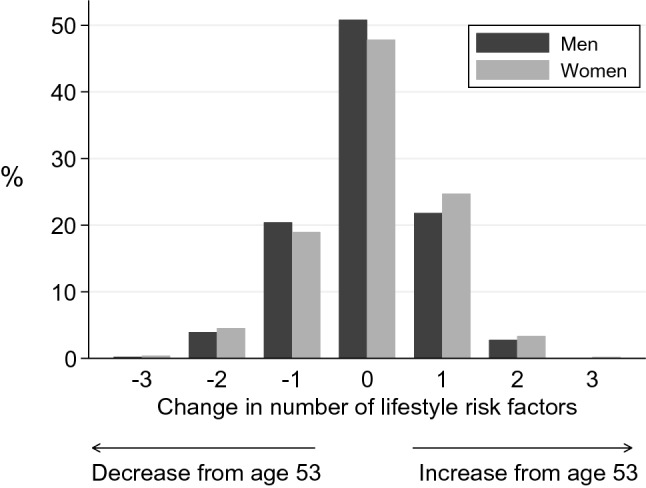
Change in number of lifestyle risk factors between age 53 and 60–64 years. Change in the number of lifestyle risk factors out of: obesity (BMI ≥ 30 kg/m^2^); inactivity (no leisure time physical activity over the previous month); current smoking; poor diet (diet quality score in bottom quarter of distribution defined at 60–64)

Standing balance time was positively skewed and was therefore log-transformed after adding one. Sex-specific standard deviation scores were coded for the physical performance outcomes to enable comparison of effect sizes. Linear regression was used to examine the cross-sectional associations between the presence versus absence of each lifestyle risk factor (obesity, inactivity, smoking, poor diet), and the total number of risk factors at age 60–64, and physical performance outcomes; associations were also examined with risk factors assessed at age 53. As some lifestyle risk factors differed between men and women (Cooper et al. 2016; Robinson et al. 2018) potential sex-lifestyle interactions were examined.

Conditional change in grip strength, chair rise speed and standing balance time from age 53 to 60–64 was characterised by residuals obtained after estimating sex-specific linear regression models for each physical performance measure at age 60–64 on the same measure at age 53, with adjustment for individual follow-up duration; these change measures are independent of baseline level (Twisk 2003). Linear regression was used to examine change in number of lifestyle risk factors between ages 53 and 60–64 in relation to levels of these outcomes at age 60–64 and to conditional changes in grip strength, chair rise speed and standing balance time from age 53 to 60–64. Models evaluating change in number of risk factors were adjusted for sex and the number of risk factors at age 53; fully adjusted models also accounted for age, follow-up time, height, diabetes and cardiovascular disease history (all ascertained at age 60–64) (Robinson et al. 2018). Analyses were conducted using Stata, release 15.

## Results

The characteristics of the participants studied are presented in Table 1. Compared to the participants who were followed up at age 60–64, but who were not included in the analyses, they had a lower prevalence of obesity, inactivity, current smoking and poor diet quality (all *p* < 0.004, Supplementary Table 1). Their mean standing balance time was greater (men and women, *p* < 0.01) and grip strength was higher (men, *p* = 0.009); chair rise speed did not differ (*p* > 0.1).

**Table 1:** Characteristics of participants in the MRC National Survey of Health and Development at ages 53 and 60–64 years

	Men (*n* = 431)		Women (n=506)	
	53 years	60–64 years	53 years	60–64 years
Age at clinic visit (years)^a^	53.4 (0.2)	63.3 (1.1)	53.4 (0.2)	63.3 (1.1)
Weight (kg)^a^	82.4 (12.0)	83.8 (12.6)	69.0 (12.8)	70.6 (13.0)
BMI (kg/m^2^)^a^	26.8 (3.5)	27.3 (3.9)	26.2 (4.7)	27.0 (4.8)
Currently in paid employment^b^	382 (88.6%)	288 (66.8%)	408 (80.6%)	214 (42.5%)
Ever had diabetes^b^		23 (5.3%)		22 (4.3%)
Ever had angina/MI^b^		26 (6.0%)		15 (3.0%)
*Lifestyle risk factors*^b^				
Obesity^c^	68 (15.8%)	98 (22.7%)	85 (16.8%)	115 (22.7%)
Inactivity^d^	168 (39.0%)	258 (59.9%)	204 (40.3%)	290 (57.3%)
Current smoker	55 (12.8%)	28 (6.5%)	76 (15.0%)	39 (7.7%)
Poor diet^e^	243 (56.4%)	143 (33.2%)	157 (31.0%)	92 (18.2%)
Risk factor categories^f^				
0	119 (27.6%)	99 (23.0%)	184 (36.4%)	160 (31.6%)
1	141 (32.7%)	177 (41.1%)	173 (34.2%)	199 (39.3%)
2	124 (28.8%)	117 (27.1%)	103 (20.4%)	109 (21.5%)
3	43 (10.0%)	36 (8.4%)	41 (8.1%)	33 (6.5%)
4	4 (0.9%)	2 (0.5%)	5 (1.0%)	5 (1.0%)
Total number of risk factors^g^	1.0 (0.0,2.0)	1.0 (1.0,2.0)	1.0 (0.0,2.0)	1.0 (0.0,2.0)
*Measures of physical performance*				
Grip strength (kg)^a^	48.2 (12.2)	45.7 (11.7)	28.6 (7.1)	26.4 (7.3)
Chair rise speed (stands/min)^a^	32.3 (10.3)	26.7 (7.1)	31.4 (9.3)	25.9 (7.4)
Standing balance time (secs)^g^	6.0 (3.0,11.0)	3.9 (2.6,5.7)	5.0 (3.0,8.0)	3.5 (2.5,5.3)

^a^Mean (standard deviation); ^b^[N(%)]; ^c^BMI ≥ 30 kg/m^2^; ^d^No leisure time physical activity over the previous month; ^e^Diet score in bottom quarter of the distribution defined at 60–64; ^f^Number of risk factors out of obesity, inactivity, current smoking and poor diet; ^g^Median (interquartile range); MI: Myocardial infarction. Sample restricted to those with complete data on lifestyle risk factors, physical performance outcomes and potential confounders. Differences between age 53 and 60–64 were statistically significant (*p* < 0.001) for all participant characteristics, both among men and women

The mean (SD) time between assessments was 9.9 (1.1) years. Over this period, the working status of many participants changed; more than 80% were in paid employment at age 53, falling to 67% of men and 43% of women at 60–64 years. Between assessments there were some changes in the pattern of lifestyle risk factors, with increases in the numbers of participants categorised as obese or inactive, alongside a fall in the prevalence of smoking, and relatively fewer participants categorised as having poor diets by age 60–64, such that the median number of lifestyle risk factors for the group did not change (Table 1). Figure 1 shows the distribution of changes in the number of lifestyle risk factors between age 53 and 60–64. Almost half (*n* = 461, 49%) had the same number of risk factors at both assessments; 227 (24%) participants had fewer by age 60–64, whilst 249 (27%) had more. The pattern was similar for men and women (Fig. 1). For the participants who had the same number of lifestyle risk factors at both assessments, the majority (*n* = 362, 79%), amounting to 39% of the whole group, had identical profiles of risk factors at both ages (data not shown).

Data on measured physical performance are shown in Table 1. As described previously in this cohort (Cooper et al. 2016), mean physical performance levels declined over the 10-year period–with reductions in mean grip strength, chair rise speed and standing balance times seen both in men and women. The associations between each of the four lifestyle risk factors assessed at the ages of 53 and 60–64, and the three measures of physical performance at 60–64, are presented in Table 2. There was little evidence of interactions between lifestyle risk factors and sex (data not shown); men and women were therefore pooled for all subsequent analyses. In the cross-sectional analyses, with the exception of smoking (chair rise speed, standing balance time) and obesity (grip strength), the presence of each lifestyle risk factor was associated with poorer physical performance at age 60–64. There were similar patterns of associations with the individual lifestyle factors that were assessed at age 53, although this was less consistent. However, a greater number of lifestyle risk factors assessed at both ages was associated with poorer physical performance at 60–64; these associations were consistent for all outcomes, and robust to adjustment for age, height and cardio-metabolic health indicators. Further detail on the changes in profiles of individual lifestyle risk factors between 53 and 60–64 is given in Supplementary Table 2; associations with the physical performance outcomes at age 60–64 are shown for participants who are categorised according to the presence of each risk factor: at neither age, at age 53, at age 60–64 or at both age 53 and 60–64. The most consistent pattern of associations was observed when lifestyle risk factors were present at both ages; for example, being inactive and having a poor diet both at 53 and at 60–64 were associated with all outcomes: poorer grip strength, chair rise speed and standing balance time (Supplementary Table 2).

**Table 2:** SD difference in physical performance measures at age 60–64 years for the presence vs absence of each lifestyle risk factor, and per unit increase in the total number, at age 53 and 60–64 years

Outcome	Age (years)	Risk factor	Sex-adjusted		Fully-adjusted^a^	
			Estimate (95% CI)	*P*-value	Estimate (95% CI)	*P*-value
Grip strength	53	Obesity^b^	− 0.09 (− 0.26,0.08)	0.304	0.00 (− 0.17,0.17)	0.978
		Inactivity^c^	− 0.30 (− 0.43,− 0.17)	< 0.001	− 0.25 (− 0.38,− 0.13)	< 0.001
		Current smoker	− 0.12 (− 0.30,0.07)	0.207	− 0.05 (− 0.23,0.12)	0.549
		Poor diet^d^	− 0.22 (− 0.36,− 0.09)	0.001	− 0.13 (− 0.26,0.00)	0.051
		Total (0–4)^e^	− 0.15 (− 0.22,− 0.09)	< 0.001	− 0.10 (− 0.16,− 0.04)	0.002
	60–64	Obesity^b^	− 0.16 (− 0.31,− 0.00)	0.046	− 0.05 (− 0.20,0.10)	0.497
		Inactivity^c^	− 0.24 (− 0.37,− 0.11)	< 0.001	− 0.20 (− 0.33,− 0.08)	0.002
		Current smoker	− 0.49 (− 0.74,− 0.25)	< 0.001	− 0.42 (− 0.66,− 0.18)	0.001
		Poor diet^d^	− 0.25 (− 0.39,− 0.10)	0.001	− 0.17 (− 0.31,− 0.02)	0.022
		Total (0–4)^e^	− 0.19 (− 0.26,− 0.12)	< 0.001	− 0.14 (− 0.21,− 0.07)	< 0.001
Chair rise speed	53	Obesity^b^	− 0.31 (− 0.48,− 0.14)	< 0.001	− 0.29 (− 0.47,− 0.11)	0.001
		Inactivity^c^	− 0.33 (− 0.46,− 0.20)	< 0.001	− 0.34 (− 0.47,− 0.21)	< 0.001
		Current smoker	− 0.22 (− 0.41,− 0.04)	0.019	− 0.24 (− 0.42,− 0.05)	0.012
		Poor diet^d^	− 0.25 (− 0.38,− 0.11)	< 0.001	− 0.25 (− 0.39,− 0.12)	< 0.001
		Total (0–4)^e^	− 0.21 (− 0.27,− 0.14)	< 0.001	− 0.22 (− 0.28,− 0.15)	< 0.001
	60–64	Obesity^b^	− 0.33 (− 0.48,− 0.17)	< 0.001	− 0.33 (− 0.48,− 0.18)	< 0.001
		Inactivity^c^	− 0.31 (− 0.44,− 0.18)	< 0.001	− 0.31 (− 0.44,− 0.18)	< 0.001
		Current smoker	− 0.21 (− 0.46,0.04)	0.100	− 0.24 (− 0.48,0.01)	0.062
		Poor diet^d^	− 0.24 (− 0.39,− 0.09)	0.002	− 0.24 (− 0.39,− 0.09)	0.002
		Total (0–4)^e^	− 0.22 (− 0.29,− 0.16)	< 0.001	− 0.23 (− 0.30,− 0.16)	< 0.001
Standing balance time	53	Obesity^b^	− 0.40 (− 0.57,− 0.23)	< 0.001	− 0.45 (− 0.63,− 0.28)	< 0.001
		Inactivity^c^	− 0.06 (− 0.19,0.07)	0.370	− 0.08 (− 0.21,0.05)	0.220
		Current smoker	− 0.05 (− 0.24,0.13)	0.591	− 0.06 (− 0.24,0.13)	0.553
		Poor diet^d^	− 0.24 (− 0.37,− 0.11)	< 0.001	− 0.26 (− 0.40,− 0.13)	< 0.001
		Total (0–4)^e^	− 0.13 (− 0.20,− 0.07)	< 0.001	− 0.15 (− 0.22,− 0.09)	< 0.001
	60–64	Obesity^b^	− 0.38 (− 0.53,− 0.22)	< 0.001	− 0.38 (− 0.53,− 0.23)	< 0.001
		Inactivity^c^	− 0.29 (− 0.42,− 0.16)	< 0.001	− 0.27 (− 0.39,− 0.14)	< 0.001
		Current smoker	− 0.02 (− 0.27,0.23)	0.884	− 0.02 (− 0.27,0.22)	0.848
		Poor diet^d^	− 0.27 (− 0.42,− 0.12)	< 0.001	− 0.29 (− 0.43,− 0.14)	< 0.001
		Total (0–4)^e^	− 0.22 (− 0.29,− 0.15)	< 0.001	− 0.22 (− 0.29,− 0.15)	< 0.001

^a^Additionally adjusted for age, height, diabetes and cardiovascular disease history (all ascertained at 60–64 years), models for risk factors at age 53 were also adjusted for follow-up time; ^b^BMI ≥ 30 kg/m^2^; ^c^no leisure time physical activity over the previous month; ^d^diet score in bottom quarter of the distribution defined at 60–64; ^e^total number of lifestyle risk factors

The principal aim of the analyses was to examine the impact of changes in lifestyle from age 53 to 60–64 on physical performance. This was assessed firstly by examining differences in the outcomes at 60–64, and secondly, by evaluating the change in the measures of physical performance over the 10-year period, conditional on these measures at baseline (age 53). The associations between change in the total number of risk factors and both the levels and conditional changes in physical performance outcomes are presented in Table 3. In both cases, reductions in the number of lifestyle risk factors from age 53 to 60–64 were related to better physical performance—evidenced both as better measured physical performance at age 60–64 and by smaller declines in these measures over the 10-year period; these associations were robust to adjustment (*p* < 0.05 for all associations). For example, a lifestyle change between 53 and 60–64 that reduced the total number of risk factors by one, was associated with a 0.11 (95% CI: 0.03,0.20) SD increase in grip strength level at age 60–64 (equivalent to a difference of 1.3 (0.4, 2.3) kg in men and 0.8 (0.2, 1.5) kg in women) and a 0.13 (0.04,0.21) SD reduction in grip strength decline from age 53 to 60–64 (fully-adjusted analyses); corresponding effect sizes for chair rise speed were 0.14 (0.06,0.23) (equivalent to 1.0 (0.4, 1.6) stands/minute in men and 1.0 (0.4, 1.7) stands /minute in women) and a 0.15 (0.07,0.24) SD reduction in chair rise speed decline.

**Table 3:** SD difference in physical performance outcome per unit decrease in the total number of lifestyle risk factors from age 53 to 60–64 years^a^

Outcome	Model	Level of outcome at age 60–64		Conditional change in outcome from age 53 to 60–64	
		Estimate (95% CI)	*P*-value	Estimate (95% CI)	*P*-value
Grip strength	1	0.14 (0.06,0.23)	0.001	0.14 (0.05,0.22)	0.001
	2	0.11 (0.03,0.20)	0.006	0.13 (0.04,0.21)	0.003
Chair rise speed	1	0.14 (0.06,0.22)	0.001	0.15 (0.07,0.24)	< 0.001
	2	0.14 (0.06,0.23)	0.001	0.15 (0.07,0.24)	< 0.001
Standing balance time	1	0.21 (0.12,0.29)	< 0.001	0.16 (0.08,0.25)	< 0.001
	2	0.19 (0.11,0.28)	< 0.001	0.18 (0.09,0.26)	< 0.001

^a^The total number of lifestyle risk factors out of: obesity (BMI ≥ 30 kg/m^2^); inactivity (no leisure time physical activity over the previous month); current smoking; poor diet (diet quality score in the bottom quarter of the distribution defined at age 60–64). A positive regression coefficient for conditional change reflects reduced decline over time (independent of level at age 53) and a negative coefficient reflects greater decline

Model 1: Adjusted for sex and the number of risk factors at age 53 years

Model 2: Additionally adjusted for age, follow-up time, height, diabetes and cardiovascular disease history (ascertained at 60–64 years)

## Discussion

Using data from a longitudinal study, in which participants were characterized in detail at the ages of 53 and 60–64, we examined differences in their total number of lifestyle risk factors (out of obesity, inactivity, smoking, poor diet) in relation to measures of physical performance in early older age. Having a greater number of lifestyle risk factors at either age was associated with poorer performance in all three measures (grip strength, chair rise speed, standing balance time) at age 60–64; these associations were robust to adjustment for covariates that included age, height and indicators of cardio-metabolic health. However, the primary focus of our study was on changes in the participants’ lifestyles that impacted on their number of risk factors, and insights these changes might provide for opportunities to promote health in the period around typical retirement age. There were two key findings: firstly that changes in the participants’ number and/or profile of risk factors over the period from age 53 were common (61% had different profiles at age 53 and 60–64), and secondly that lifestyle change to reduce the number of risk factors over this time was associated with better physical performance at age 60–64. This was a consistent finding for all outcome measures, and robust to adjustment for covariates.

In recent years, evidence of links between lifestyle and physical performance has grown. But, as health behaviours are known to cluster (Poortinga 2007) a clearer understanding of their combined effects is needed. This approach has been used to evaluate effects of lifestyle on a range of health outcomes, including mortality, cardiovascular disease and cancer (Barbaresko et al. 2018; Liao et al. 2018; Zhang et al. 2020) but in comparison, less attention has been given to physical performance. The studies published to date have mainly included older populations (Koster et al. 2007; Liao et al. 2011; Robinson et al. 2013; Visser et al. 2019) which limits comparisons with our findings. However, there are two studies that are very relevant, from an earlier analysis of data from the same cohort (Cooper et al. 2016) and a longitudinal study of women that started in mid-life (Sternfeld et al. 2017). In the first of these studies, changes in grip strength and chair rise speed between 53 and 60–64 were examined in relation to a risk factor count (out of obesity, inactivity and smoking); a greater count at age 53 was associated with poorer measures of these outcomes and with an increased risk of decline with age. The present analyses add to these findings by considering poor diet as a further risk factor (Robinson et al. 2018), including a third measure of physical performance (standing balance) and evaluating the impact of changes in these lifestyle risk factors over time. The associations with standing balance are consistent with the earlier findings for other outcomes (Cooper et al. 2016) showing improved performance among participants who had fewer lifestyle risk factors. However, the present study showed for the first time, that the participants who had changed their lifestyles, to have fewer risk factors by age 60–64, had better physical performance. This was also evident in conditional models that took account of baseline measures, suggesting lifestyle change to reduce risk factors has protective benefits, slowing declines in physical performance over time.

In the second study, Sternfeld and colleagues described differences in measured physical performance of 1769 women in the SWAN study, aged 56–68 years, in association with an averaged healthy lifestyle score, based on physical activity, smoking, and diet in the period from baseline (ages 42–52). The age group was therefore comparable to the present study, and findings for some of the performance measures in relation to lifestyle were consistent (positive effects on walking speed, chair rise speed). However, there are differences in findings between the studies. In our analyses, the number of lifestyle factors was related to all physical performance measures, before and after adjustment for covariates; in contrast, in the SWAN study there were no associations with grip strength or with standing balance tests in the adjusted models (Sternfeld et al. 2017). These differences may be explained by differences in the covariates included in the models, but additionally, may be due to inclusion of obesity in the risk factor count in our study, whereas this was used to stratify analyses in the SWAN study.

To our knowledge, no other studies have evaluated the impact of combined changes in lifestyle risk factors on physical performance in early older age. We found that changes in lifestyle between age 53 and 60–64 years were common: obesity and inactivity were more prevalent at follow-up, although these increases were balanced by other positive changes (reduction in smoking, fewer participants with low quality diets). The change in diet was expected, as we have previously described increases in diet quality across adulthood in this cohort (Robinson et al. 2018), and the observed difference in other lifestyle risk factors are in line with data from the wider population that indicate comparable age-related differences and secular trends across the period between 1999 and 2010 (NatCen Social Research 2014) around the time the participants were assessed. Our findings should therefore have wider relevance beyond this cohort. In population studies, grip strength and chair rise speed have been shown to be predictive of poorer health outcomes, even when measured in middle aged adults (Cooper et al. 2010; Dodds et al. 2018). The observed differences in these outcomes in relation to a change in the number of risk factors between 53 and 60–64, although modest, should therefore have clinical relevance. For example meta-analysis of grip strength data has shown a 1 kg difference at baseline to predict lower mortality over follow-up (Cooper et al. 2010; Rijk et al 2016); the difference in grip strength associated with a change in one risk factor in our analyses was 1.3 (0.4, 2.3) kg in men and 0.8 (0.2, 1.5) kg in women.

Our primary aim was to examine the potential effects of lifestyle change on physical performance, examining changes in lifestyle risk factors in a group of men and women in the period around typical retirement age. Although there were significant changes in the proportion of participants who were in employment, from more than 80% (men and women) at age 53 to 67% (men) and 43% (women) at 60–64 years, which are likely to have impacted on the changes in lifestyle that we observed, our analyses did not address the effects of retiring from work directly. A number of studies have described changes in lifestyle following retirement although overall messages regarding the nature of these changes are mixed, depending in part on preceding occupation and reasons for retirement as well as other personal characteristics, such as gender (Baxter et al. 2016; Myllyntausta et al. 2017; Pulakka et al. 2019; Ali-Kovero et al 2020). Whilst this study does not address retirement effects, it does provide encouraging evidence that lifestyle change is possible around this age, pointing both to the potential for health benefits of positive changes in health behaviours and to opportunity for intervention to support healthier lifestyles in early older age. This potential is further highlighted by the finding that the most consistent pattern of associations with poorer physical performance was seen among participants who had not changed lifestyle, and who had risk factors at both ages.

Strengths of our study include the detailed lifestyle data collected prospectively from the study participants, and the availability of repeat data on physical performance that was assessed by trained research nurses using standardized protocols (Kuh et al. 2011). However, it is a weakness that our analyses were limited to a subsample of the cohort for whom there were complete data. Participants in this group had fewer lifestyle risk factors and better physical performance when compared to others who were excluded, although as our analyses were based on internal comparisons, bias should only be introduced if the associations reported differed systematically among those who were and were not included. Importantly, by focussing only on participants who had paired assessments of lifestyle and physical performance at ages of 53 and 60–64, we were able to evaluate the effects of lifestyle change. Additionally, using a residual change method we could examine changes in physical performance that were independent of baseline levels and free from the effect of regression to the mean, although as we did not have information on the determinants of lifestyle change, we cannot exclude the possibility that reverse causation or residual confounding could have contributed to the associations observed. In particular, participants’ explanations for a lack of change in their activity levels and/or changes in obesity status between 53 and 60–64 years would be of relevance. A further limitation was the use of dichotomous variables for each lifestyle risk factor that were summed to form a risk factor score. We took this approach to provide a simple summary of lifestyle behaviours, but it meant that more detailed information on the continuous measures available (BMI and diet quality) was not utilised.

## Conclusion

Our findings suggest that reducing the number of lifestyle risk factors in the period around typical retirement age has beneficial effects, such that lifestyle change may have significant potential as a way to improve physical performance in early older age. Although evidence of adherence to health recommendations among retired adults is mixed (King and Xiang 2017) there may be particular opportunities at retirement transition when lifestyle interventions would be effective (Lara et al. 2014; O'Brien et al. 2015). Our observational findings need confirmation in other studies, but could have important public health implications.

## Supplementary information

Below is the link to the electronic supplementary material.

Supplementary information 1 (DOCX 95 kb)

## References

[CR1] Ali-Kovero K, Pietilainen O, Mauramo E, Jappinen S, Rahkonen O, Lallukka T (2020). Changes in fruit, vegetable and fish consumption after statutory retirement: a prospective cohort study. Br J Nutr.

[CR2] Barbaresko J, Rienks J, Nothlings U (2018). Lifestyle Indices and Cardiovascular Disease Risk: A Meta-analysis. Am J Prev Med.

[CR3] Baxter S, Johnson M, Payne N, Buckley-Woods H, Blank L, Hock E (2016). Promoting and maintaining physical activity in the transition to retirement: a systematic review of interventions for adults around retirement age. Int J Behav Nutr Phys Act.

[CR4] Buck D, Frosini F (2012) Clustering of unhealthy behaviours over time. Implications for policy and practice. Report of the King's Fund [online]. http://www.kingsfund.org.uk/publications/unhealthy_behaviours.html.

[CR5] Cooper R, Kuh D, Hardy R, Mortality Review Group FALcon and HALCyon Team (2010). Objectively measured physical capability levels and mortality: systematic review and meta-analysis. BMJ.

[CR6] Cooper R, Mishra GD, Kuh D (2011). Physical activity across adulthood and physical performance in midlife: findings from a British birth cohort. Am J Prev Med.

[CR7] Cooper R, Muniz-Terrera G, Kuh D (2016). Associations of behavioural risk factors and health status with changes in physical capability over 10 years of follow-up: the MRC National Survey of Health and Development. BMJ Open.

[CR8] Dodds R, Kuh D, Aihie Sayer A, Cooper R (2013). Physical activity levels across adult life and grip strength in early old age: updating findings from a British birth cohort. Age Ageing.

[CR9] Dodds RM, Kuh D, Sayer AA, Cooper R (2018). Can measures of physical performance in mid-life improve the clinical prediction of disability in early old age? Findings from a British birth cohort study. Exp Gerontol.

[CR10] England K, Azzopardi-Muscat N (2017). Demographic trends and public health in Europe. Eur J Public Health.

[CR11] Hurst L, Stafford M, Cooper R, Hardy R, Richards M, Kuh D (2013). Lifetime socioeconomic inequalities in physical and cognitive aging. Am J Public Health.

[CR12] King DE, Xiang J (2017). Retirement and Healthy Lifestyle: A National Health and Nutrition Examination Survey (NHANES) Data Report. J Am Board Fam Med.

[CR13] Koster A, Penninx BW, Newman AB, Visser M, van Gool CH, Harris TB (2007). Lifestyle factors and incident mobility limitation in obese and non-obese older adults. Obesity (Silver Spring).

[CR14] Kuh D, Pierce M, Adams J, Deanfield J, Ekelund U, Friberg P (2011). Cohort profile: updating the cohort profile for the MRC National Survey of Health and Development: a new clinic-based data collection for ageing research. Int J Epidemiol.

[CR15] Lara J, Hobbs N, Moynihan PJ, Meyer TD, Adamson AJ, Errington L (2014). Effectiveness of dietary interventions among adults of retirement age: a systematic review and meta-analysis of randomized controlled trials. BMC Med.

[CR16] Liao J, Muniz-Terrera G, Scholes S, Hao Y, Chen YM (2018). Lifestyle index for mortality prediction using multiple ageing cohorts in the USA. UK and Europe Sci Rep.

[CR17] Liao WC, Li CR, Lin YC, Wang CC, Chen YJ, Yen CH (2011). Healthy behaviors and onset of functional disability in older adults: results of a national longitudinal study. J Am Geriatr Soc.

[CR18] Lowsky DJ, Olshansky SJ, Bhattacharya J, Goldman DP (2014). Heterogeneity in healthy aging. J Gerontol A Biol Sci Med Sci.

[CR19] McGinnis JM, Williams-Russo P, Knickman JR (2002). The case for more active policy attention to health promotion. Health Aff (Millwood).

[CR20] Newton JN, Briggs AD, Murray CJ, Dicker D, Foreman KJ, Wang H (2015). Changes in health in England, with analysis by English regions and areas of deprivation, 1990–2013: a systematic analysis for the Global Burden of Disease Study 2013. Lancet.

[CR21] O'Brien N, McDonald S, Araujo-Soares V, Lara J, Errington L, Godfrey A (2015). The features of interventions associated with long-term effectiveness of physical activity interventions in adults aged 55–70 years: a systematic review and meta-analysis. Health Psychol Rev.

[CR22] Poortinga W (2007). The prevalence and clustering of four major lifestyle risk factors in an English adult population. Prev Med.

[CR23] Pot GK, Prynne CJ, Almoosawi S, Kuh D, Stephen AM (2015). Trends in food consumption over 30 years: evidence from a British birth cohort. Eur J Clin Nutr.

[CR24] Myllyntausta S, Salo P, Kronholm E, Aalto V, Kivimaki M, Vahtera J (2017). Changes in sleep duration during transition to statutory retirement: a longitudinal cohort study. Sleep.

[CR25] NatCen Social Research, University College London. Department of Epidemiology and Public Health. (2014). Health Survey for England, 2012. https://digital.nhs.uk/data-and-information/publications/statistical/health-survey-for-england/health-survey-for-england-2012#resources

[CR26] Pulakka A, Halonen JI, Pentti J, Kivimaki M, Vahtera J, Stenholm S (2019). Changes in smoking during retirement transition: a longitudinal cohort study. Scand J Public Health.

[CR27] Rijk JM, Roos PR, Deckx L, van den Akker M, Buntinx F (2016). Prognostic value of handgrip strength in people aged 60 years and older: A systematic review and meta-analysis. Geriatr Gerontol Int.

[CR28] Rizzuto D, Fratiglioni L (2014). Lifestyle factors related to mortality and survival: a mini-review. Gerontology.

[CR29] Robinson SM, Jameson KA, Syddall HE, Dennison EM, Cooper C, Aihie Sayer A (2013). Clustering of lifestyle risk factors and poor physical function in older adults: The Hertfordshire Cohort Study. J Am Geriatr Soc.

[CR30] Robinson SM, Westbury LD, Cooper R, Kuh D, Ward K, Syddall HE (2018). Adult Lifetime Diet Quality and Physical Performance in Older Age: Findings from a British Birth Cohort. J Gerontol A Biol Sci Med Sci.

[CR31] Sabia S, Singh-Manoux A, Hagger-Johnson G, Cambois E, Brunner EJ, Kivimaki M (2012). Influence of individual and combined healthy behaviours on successful aging. CMAJ.

[CR32] Stenholm S, Tiainen K, Rantanen T, Sainio P, Heliovaara M, Impivaara O, Koskinen S (2012). Long-term determinants of muscle strength decline: prospective evidence from the 22-year mini-Finland follow-up survey. J Am Geriatr Soc.

[CR33] Sternfeld B, Colvin A, Stewart A, Dugan S, Nackers L, El Khoudary SR (2017). The Effect of a Healthy Lifestyle on Future Physical Functioning in Midlife Women. Med Sci Sports Exerc.

[CR34] Twisk JWR (2003). Applied longitudinal data analysis for epidemiology : a practical guide.

[CR35] Visser M, Wijnhoven HAH, Comijs HC, Thomese F, Twisk JWR, Deeg DJH (2019). A Healthy Lifestyle in Old Age and Prospective Change in Four Domains of Functioning. J Aging Health.

[CR36] Zantinge EM, van den Berg M, Smit HA, Picavet HS (2014). Retirement and a healthy lifestyle: opportunity or pitfall? A narrative review of the literature. Eur J Public Health.

[CR37] Zhang YB, Pan XF, Chen J, Cao A, Zhang YG, Xia L (2020). Combined lifestyle factors, incident cancer, and cancer mortality: a systematic review and meta-analysis of prospective cohort studies. Br J Cancer.

