# Supplementary material for: Is lifestyle change around retirement associated with better physical performance in older age?: insights from a longitudinal cohort
Source: Eur J Ageing. 2021 May 27;18(4):513–21. doi: 10.1007/s10433-021-00607-9 (PMC8563887; doi:10.1007/s10433-021-00607-9)
Supplement: Supplementary file 1 — Supplementary information 1 (DOCX 95 kb) [file 10433_2021_607_MOESM1_ESM.docx]

| **Supplementary Table 1: Comparison of lifestyle risk factors and physical performance outcomes at 60-64 years between the analysis sample (n=937) and those with incomplete data who were excluded (n=1292)** | | | | | | |
| --- | --- | --- | --- | --- | --- | --- |
|  |  |  |  |  |  |  |
|  | **Men (n=1067)** | | | **Women (n=1162)** | | |
|  | **Included (n=431)** | **Excluded (n=636)** | **P-value** | **Included (n=506)** | **Excluded (n=656)** | **P-value** |
| **Lifestyle risk factors^b^** |  |  |  |  |  |  |
| Obesity^c^ | 98 (22.7%) | 199 (31.6%) | 0.002 | 115 (22.7%) | 236 (36.3%) | <0.001 |
| Inactivity^d^ | 258 (59.9%) | 417 (68.8%) | 0.003 | 290 (57.3%) | 415 (67.0%) | 0.001 |
| Current smoker | 28 (6.5%) | 80 (14.8%) | <0.001 | 39 (7.7%) | 81 (14.7%) | <0.001 |
| Poor diet^e^ | 143 (33.2%) | 213 (47.7%) | <0.001 | 92 (18.2%) | 132 (27.4%) | 0.001 |
|  |  |  |  |  |  |  |
| **Measures of physical performance** | |  |  |  |  |  |
| Grip strength (kg)^a^ | 45.7 (11.7) | 43.7 (11.6) | 0.009 | 26.4 (7.3) | 25.6 (7.5) | 0.113 |
| Chair rise speed (stands/min)^a^ | 26.7 (7.1) | 26.2 (7.4) | 0.237 | 25.9 (7.4) | 25.1 (8.5) | 0.128 |
| Standing balance time (secs)^f^ | 3.9 (2.6,5.7) | 3.5 (2.3, 5.6) | 0.009 | 3.5 (2.5,5.3) | 3.2 (2.1, 4.6) | <0.001 |
| ^a^Mean (standard deviation); ^b^[N(%)]; ^c^BMI ≥ 30kg/m^2^; ^d^No leisure time physical activity over the previous month; ^e^Diet score in bottom quarter of the distribution defined at 60-64; ^f^Median (interquartile range); Analysis sample restricted to those with complete data on lifestyle risk factors, physical performance outcomes and potential confounders; P-values presented for the difference in characteristics within each sex between those who were included in the analysis sample and those who were excluded | | | | | | |

| **Supplementary Table 2: SD difference in physical performance measures at age 60-64 years according to each risk factor profile** | | | | | | | | | |  |  |  |
| --- | --- | --- | --- | --- | --- | --- | --- | --- | --- | --- | --- | --- |
|  |  |  |  |  |  |  |  |  |  |  |  |  |
|  | **Grip strength** | | | | **Chair speed** | | | | **Standing balance time** | | | |
| **Presence at 53 and 60-64 years** | **Sex-adjusted** | | **Fully-adjusted^a^** | | **Sex-adjusted** | | **Fully-adjusted^a^** | | **Sex-adjusted** | | **Fully-adjusted^a^** | |
|  | **Estimate** | **P-value** | **Estimate** | **P-value** | **Estimate** | **P-value** | **Estimate** | **P-value** | **Estimate** | **P-value** | **Estimate** | **P-value** |
|  | **(95% CI)** |  | **(95% CI)** |  | **(95% CI)** |  | **(95% CI)** |  | **(95% CI)** |  | **(95% CI)** |  |
| **Obesity^b^** |  |  |  |  |  |  |  |  |  |  |  |  |
| Neither (74.5%) | *Reference category* | |  |  |  |  |  |  |  |  |  |  |
| 53 only (2.8%) | 0.16 (-0.23,0.56) | 0.410 | 0.26 (-0.12,0.64) | 0.173 | -0.06 (-0.45,0.33) | 0.758 | -0.02 (-0.42,0.37) | 0.901 | -0.53 (-0.92,-0.14) | 0.007 | -0.58 (-0.96,-0.19) | 0.003 |
| 60-64 only (9.2%) | -0.13 (-0.36,0.09) | 0.246 | -0.02 (-0.24,0.20) | 0.856 | -0.24 (-0.46,-0.02) | 0.035 | -0.26 (-0.48,-0.04) | 0.021 | -0.36 (-0.58,-0.13) | 0.002 | -0.31 (-0.53,-0.09) | 0.005 |
| Both (13.6%) | -0.16 (-0.35,0.03) | 0.096 | -0.05 (-0.24,0.13) | 0.578 | -0.39 (-0.58,-0.20) | <0.001 | -0.38 (-0.57,-0.19) | <0.001 | -0.42 (-0.61,-0.24) | <0.001 | -0.47 (-0.66,-0.28) | <0.001 |
|  |  |  |  |  |  |  |  |  |  |  |  |  |
| **Inactivity^c^** |  |  |  |  |  |  |  |  |  |  |  |  |
| Neither (32.4%) | *Reference category* | |  |  |  |  |  |  |  |  |  |  |
| 53 only (9.1%) | -0.08 (-0.32,0.15) | 0.493 | -0.05 (-0.28,0.17) | 0.637 | -0.15 (-0.38,0.09) | 0.225 | -0.15 (-0.39,0.08) | 0.208 | 0.13 (-0.11,0.37) | 0.275 | 0.10 (-0.13,0.34) | 0.398 |
| 60-64 only (27.9%) | -0.08 (-0.25,0.08) | 0.315 | -0.06 (-0.22,0.09) | 0.428 | -0.18 (-0.35,-0.02) | 0.027 | -0.17 (-0.33,-0.01) | 0.040 | -0.25 (-0.42,-0.09) | 0.002 | -0.22 (-0.38,-0.06) | 0.008 |
| Both (30.6%) | -0.42 (-0.57,-0.26) | <0.001 | -0.35 (-0.50,-0.20) | <0.001 | -0.49 (-0.65,-0.33) | <0.001 | -0.49 (-0.65,-0.34) | <0.001 | -0.27 (-0.43,-0.11) | 0.001 | -0.27 (-0.43,-0.11) | 0.001 |
|  |  |  |  |  |  |  |  |  |  |  |  |  |
| **Current smoker** |  |  |  |  |  |  |  |  |  |  |  |  |
| Neither (85.5%) | *Reference category* | |  |  |  |  |  |  |  |  |  |  |
| 53 only (7.4%) | 0.20 (-0.04,0.45) | 0.104 | 0.26 (0.02,0.49) | 0.032 | -0.14 (-0.38,0.11) | 0.268 | -0.15 (-0.39,0.10) | 0.239 | -0.08 (-0.33,0.17) | 0.531 | -0.09 (-0.33,0.16) | 0.480 |
| 60-64 only (0.5%) | -0.41 (-1.28,0.47) | 0.361 | -0.31 (-1.15,0.53) | 0.468 | 0.80 (-0.08,1.68) | 0.073 | 0.74 (-0.13,1.61) | 0.097 | -0.08 (-0.96,0.80) | 0.861 | -0.14 (-1.01,0.72) | 0.744 |
| Both (6.6%) | -0.48 (-0.74,-0.23) | <0.001 | -0.40 (-0.65,-0.16) | 0.001 | -0.30 (-0.56,-0.04) | 0.021 | -0.33 (-0.58,-0.07) | 0.013 | -0.02 (-0.28,0.24) | 0.877 | -0.02 (-0.28,0.23) | 0.865 |
|  |  |  |  |  |  |  |  |  |  |  |  |  |
| **Poor diet^d^** |  |  |  |  |  |  |  |  |  |  |  |  |
| Neither (52.7%) | *Reference category* | |  |  |  |  |  |  |  |  |  |  |
| 53 only (22.2%) | -0.21 (-0.37,-0.05) | 0.012 | -0.13 (-0.29,0.03) | 0.106 | -0.23 (-0.40,-0.07) | 0.005 | -0.23 (-0.40,-0.07) | 0.005 | -0.17 (-0.34,-0.01) | 0.040 | -0.21 (-0.37,-0.04) | 0.013 |
| 60-64 only (4.6%) | -0.36 (-0.67,-0.05) | 0.024 | -0.35 (-0.65,-0.06) | 0.020 | -0.30 (-0.61,0.01) | 0.058 | -0.31 (-0.62,-0.00) | 0.047 | -0.20 (-0.51,0.11) | 0.197 | -0.24 (-0.54,0.07) | 0.127 |
| Both (20.5%) | -0.30 (-0.47,-0.13) | <0.001 | -0.18 (-0.35,-0.01) | 0.034 | -0.32 (-0.49,-0.15) | <0.001 | -0.32 (-0.49,-0.15) | <0.001 | -0.35 (-0.52,-0.18) | <0.001 | -0.39 (-0.56,-0.22) | <0.001 |
| ^a^Additionally adjusted for age, height, diabetes and cardiovascular disease history (all ascertained at 60-64 years);  ^b^BMI ≥ 30kg/m^2^; ^c^no leisure time physical activity over the previous month; ^d^diet score in bottom quarter of the distribution defined at 60-64. | | | | | | | | | | | | |

**
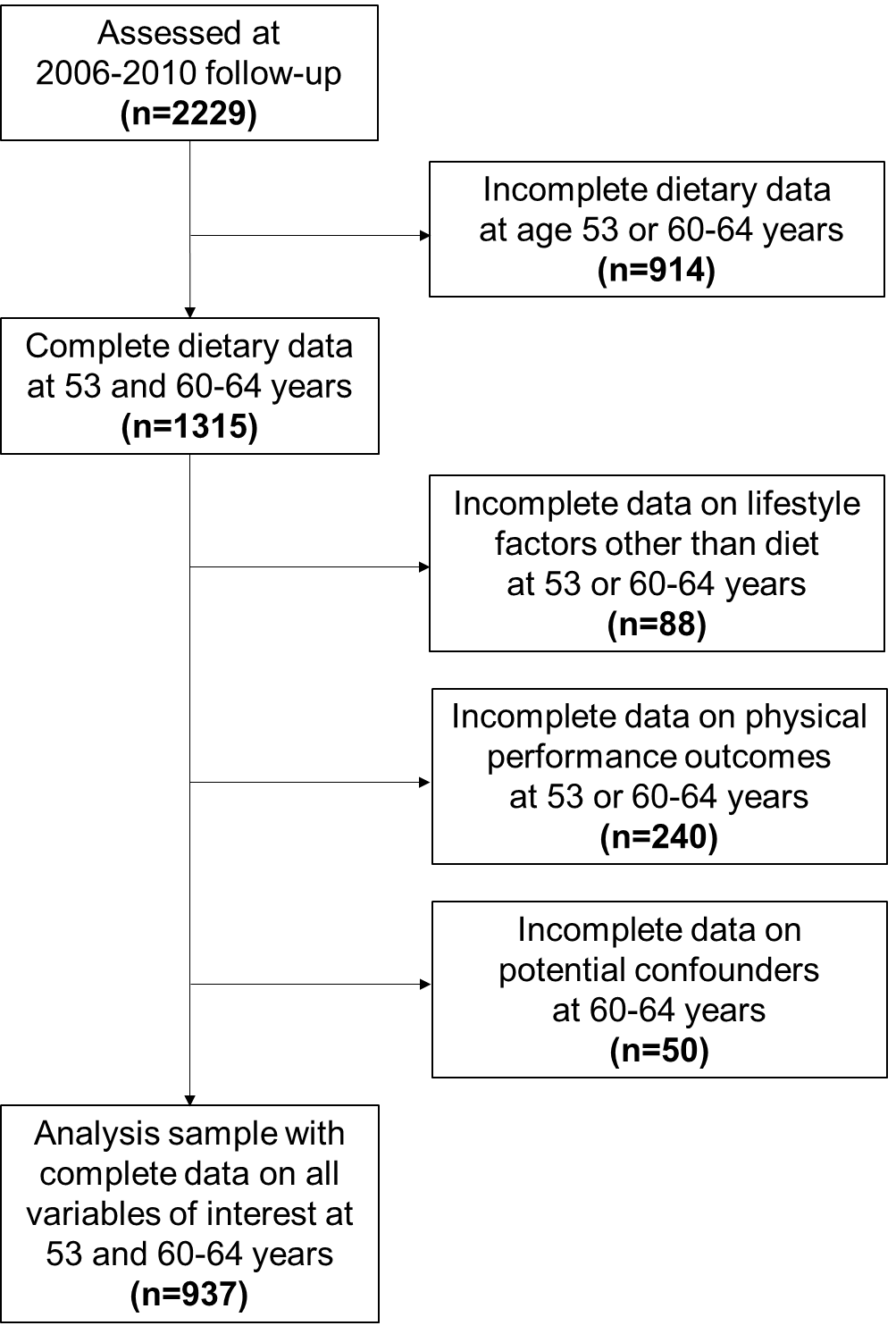
**

**Supplementary Figure 1: Flow diagram for analysis sample**
